# County-Level Life Expectancy Change: A Novel Metric for Monitoring Public Health

**DOI:** 10.3390/ijerph191710672

**Published:** 2022-08-27

**Authors:** Aruna Chandran, Ritika Purbey, Kathryn M. Leifheit, Kirsten McGhie Evans, Jocelyn Velasquez Baez, Keri N. Althoff

**Affiliations:** 1 Department of Epidemiology, Johns Hopkins Bloomberg School of Public Health, Baltimore, MD 21205, USA; 2 Department of Health Policy and Management, UCLA Fielding School of Public Health, Los Angeles, CA 90095, USA

**Keywords:** life expectancy, social determinants of health, COVID-19 mortality, health equity

## Abstract

Life expectancy (LE) is a core measure of population health. Studies have confirmed the predictive importance of modifiable determinants on LE, but less is known about their association with LE change over time at the US county level. In addition, we explore the predictive association of LE change with COVID-19 mortality. We used a linear regression model to calculate county-level annual LE change from 2011 to 2016, and categorized LE change (≤−0.1 years change per year as decreasing, ≥0.1 years as increasing, otherwise no change). A multinomial regression model was used to determine the association between modifiable determinants of health indicators from the County Health Rankings and LE change. A Poisson regression model was used to evaluate the relationship between change in life expectancy and COVID-19 mortality through September 2021. Among 2943 counties, several modifiable determinants of health were significantly associated with odds of being in increasing LE or decreasing LE counties, including adult smoking, obesity, unemployment, and proportion of children in poverty. The presence of an increasing LE in 2011–2016, as compared to no change, was significantly associated with a 5% decrease in COVID-19 mortality between 2019 and 2021 (β = 0.953, 95% CI: 0.943, 0.963). We demonstrated that change in LE at the county level is a useful metric for tracking public health progress, measuring the impact of public health initiatives, and gauging preparedness and vulnerability for future public health emergencies.

## 1. Introduction

Life expectancy (LE) is a core measure of the health of a population, capturing its physical, mental, social, and economic strength [1]. The US LE has lagged behind other high-income countries for decades; the 2019 overall LE (prior to the COVID-19 pandemic) was 78.9 years, compared to the average among comparable countries of 82.6 years [2,3]. Even more alarmingly, there is an over 20-year difference in life expectancy between the highest and lowest LE counties within the US, with inequalities increasing over time [4,5,6]. Underlying these inequities are socioeconomic, behavioral, and environmental risk factors [4].

Instead of the measurement of LE at a single time point, LE change over time can be used to measure the overall progress towards health improvements and to monitor disparities. For example, studies have found more pronounced increases in LE over time among individuals with higher educational attainment and income, and demonstrated widening gaps in LE between geographic areas in the lower education and income subgroups [7]. However, few studies have focused on using LE change or trends over time as an independent metric of community health.

LE estimates are more sensitive to deaths at younger and midlife ages compared to older ages [8]. Between 2015 and 2017, the US saw LE decline year after year for the first time in decades [9]. Demographically, this trend is explained by increased midlife deaths among white non-Hispanic Americans due to the so-called deaths of despair [10]. However, county-level heterogeneity in life expectancy trends up to and including this period has not been well-characterized. Whereas LE fell in aggregate, some communities may have improved in increasing life expectancy during this period, while others saw declines. Identifying features of communities associated with these divergent trends might help identify community-level risks and protective factors for LE changes during this transformational period. 

The County Health Rankings (CHRs), developed by the Robert Wood Johnson Foundation in collaboration with the University of Wisconsin Population Health Institute, provide estimates and within-state rankings on several health-related metrics for nearly every county in the US [11]. The rankings are based on modifiable determinants and health outcomes, including life expectancy and indicators of quality of life. Several studies confirmed the predictive importance of the chosen modifiable determinants (including socioeconomic factors, health behaviors, clinical care, and the physical environment) on core health outcomes, including LE [12]. Montez et al. showed that modifications in state-level policies in several areas, such as tobacco control and civil rights, could increase LE in some areas by over 2 years [9]. Similar explorations at the county level (the jurisdictional level at which many health-related regulations are created) are also important, but, to our knowledge, have not been performed. Furthermore, while annual LE is a core indicator included in the CHRs, LE change over time is not.

The onslaught of the COVID-19 pandemic brought about additional declines in LE. Projections suggest that from 2019 to 2020, LE dropped by 1.13 years, a ten-fold greater decrease than the decreases seen from 2015 to 2017 [13]. It is important to consider COVID-19 mortality against a backdrop of differences in declining life expectancy in US counties. However, the degree to which COVID deaths overlapped geographically with LE declines observed earlier in the decade remains unknown.

Our objectives were three-fold: (1) to estimate LE change at the county level between 2011 and 2016 and delineate counties with increasing, decreasing, or no change in LE; (2) to identify community-level predictors of LE change by measuring associations between core CHR metrics in 2010 and LE change in the subsequent 5-year period; and (3) to estimate the associations between the 2011–2016 LE change and county-level COVID-19 mortality through September 2021. In doing so, we sought to gauge the utility of county-level LE change to measure health progress, describe the impact of modifiable determinants of health in communities, and predict community vulnerability to future mortality declines.

## 2. Materials and Methods

### 2.1. Overview

The analysis was conducted in three steps, corresponding to the three objectives. We first estimated county-level LE annually from 2011 to 2016 using methods developed by the US National Center for Health Statistics (NCHS) for small-area life expectancy [14]. This method leverages demographic data to estimate LE in counties with limited age-specific mortality data. We then classified counties as having either increasing, decreasing, or stable LE change from 2011 to 2016 and described geographic patterns using LE change categories. Second, we analyzed associations between 2010 CHR metrics and 2011–2016 LE change. Third, we analyzed associations between the 2011–2016 LE change and COVID-19 mortality from January 2020 to September 2021.

### 2.2. Data Sources

To calculate life expectancies from 2011 to 2016, we obtained unsuppressed mortality data following approval for use of restricted vital statistics data from the National Center for Health Statistics, which includes information on deaths by age, sex, race, and ethnicity [15]. Population data for life expectancy calculations and regression covariates were extracted from the US Census Bureau’s American Community Survey (ACS), with ACS years varying between analyses, as noted below. Counties that include all or part of a Native American reservation, or that shared a boundary with a reservation, were identified using 2006 Purchased/Referred Care Service Delivery Area (PRCSDA) definitions from the National Cancer Institute. Data on modifiable health determinants were extracted from the 2010 Robert Wood Johnson County Health Rankings [11]. County-level COVID-19 mortality data from 1 January 2020 to 8 September 2021 were obtained from the National Center of Health Statistics [16].

### 2.3. Life Expectancy

Counties were included in the analysis if they had age-specific population data for all years of analysis (4 counties were excluded for this reason). Annual counts of deaths were aggregated to the following age groups: 0 years, 1–4 years, 5–9 years, 10–14 years, 15–19 years, 20–24 years, 25–34 years, 35–44 years, 45–54 years, 55–64 years, 65–74 years, 75–84 years, and 85 years and above. Any reported deaths with unknown age at death were excluded. The age-group-specific number of deaths for each year was calculated by averaging deaths from the year of interest and the preceding two years in order to smooth out annual variations.

For county and age stratum cells in which there were <5 observed deaths (using the three-year-averaged counts), the number of deaths was replaced with an estimated death count if the estimated death number was higher. Estimated death counts were derived using the following approach: A negative binomial regression model was fitted with the following covariates: age group, total county population, median income quartile indicators, population density quartile indicators, proportion of non-Hispanic Black quartile indicators, proportion of Hispanic quartile indicators, proportion of individuals with a 4-year college degree or higher quartile indicators, PRCSDA status, and US Census region indicators [17]. These covariates were selected to match what the NCHS does and recommends in their small-area LE calculations [14]. To confirm that our estimated death counts were consistent with known population-level trends in mortality, we derived model parameters from a subset of counties with a 2010 population over 5000, nonmissing age-specific death counts after averaging 2008–2010 deaths, and a distribution of age-specific death rates consistent with our expectations. Specifically, we confirmed that the age-specific death rate for individuals aged 0 years was higher than that for those aged 5–9, that the county’s minimum death rate was either the 5–9 year age category or the 10–14 year category, and that deaths increased more steeply between the 55–64 and 65–74 age categories than they did between the 45–54 and 65–74 age categories. These methods were similar to the small cell prediction methods used in the NCHS US small-area life expectancy report [14].

Using these counts, at-birth life expectancy for each county year (2011–2016) was estimated using the Chiang 1984 method [18]. One county was excluded because it had no individuals in some age categories. As further confirmation of our calculations, we compared and confirmed the consistency of our calculated county life expectancies to those shown in the NCHS US small-area life expectancies, aggregating the NCHS census tract estimates to the county level using population-weighted averages (data not shown).

A linear regression model was used to estimate the average change in life expectancy per year for each county. Counties were categorized as having increasing, decreasing, or no change in life expectancy between 2011 and 2016 using a categorization of 0.1 years change in life expectancy per year (i.e., a slope of ≤−0.1 years classified as decreasing, ≥0.1 years classified as increasing, and otherwise classified as no change).

### 2.4. Statistical Analysis 

Descriptive statistics were used to show summary statistics and the geographic region distribution of LE change and COVID-19 mortality for all counties included in the final analyses. A multinomial regression model was used to determine the association between indicators of modifiable determinants of health and county-level LE change (“increasing” or “decreasing” as compared to “no change” counties). Detailed definitions of the indicators of modifiable determinants are given in Supplementary Materials, Table S1. Covariates in the model included baseline 2010 life expectancy and proportions of the county ≤19 and >55 years of age based on 2011–2016 ACS estimates. Baseline life expectancy was included as a measure of the overall health status of the county prior to the period for which LE change was assessed. The proportion of the population ≤19 years of age was included as early-age deaths significantly affect LE calculations. Given the increased risk of mortality in the higher age ranges, the proportion of the population >55 years of age was also included as a covariate.

A Poisson regression model was used to evaluate the relationship between changes in life expectancy and COVID-19 mortality from January 2020 to September 2021. The model was adjusted for baseline 2010 life expectancy, age proportions ≤19 and >55 years based on the 2011–2016 ACS, unemployment derived from the 2011–2016 ACS (individuals aged ≥16 years that reported seeking work), poor physical health days from the CHRs for 2010 (average physically unhealthy days/month for adult), and population density from the 2016 ACS (calculated as number of people per square kilometer, divided into quintiles). Unemployment was included as a measure of socioeconomic status, which has been shown to be associated with COVID-19 mortality [19]. Poor physical health days were used as a proxy for underlying medical conditions, a known risk factor for COVID-19 mortality [20]. Population density was added as a covariate in this model, given the increased rates of COVID-19 transmission that occurred in highly dense populations [21]. All analyses were performed in R Version 4.0.3 and STATA version 17, and *p*-values of ≤0.05 guided statistical interpretations.

## 3. Results

### 3.1. Description of LE Change

**Figure 1:**
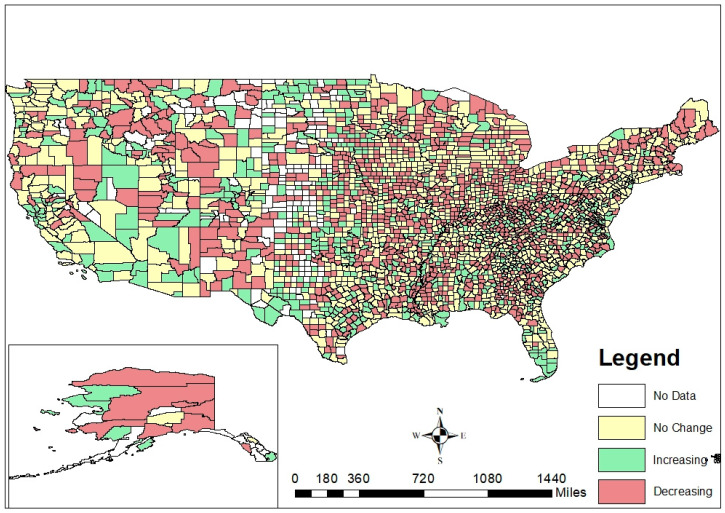
County-level life expectancy change in the United States, 2011–2016.

LE change at the county level across the US is shown in Figure 1. To test the hypothesis that the direction of LE change would not necessarily directly correlate with baseline LE, we compared the number of “increasing”, “decreasing”, and “no change” counties that fell within each tertile of baseline LE (Table 1). Of the counties in the lowest tertile of baseline LE in 2010, 26% (*n* = 256) had increasing LE between 2011 and 2016 and 48% (*n* = 472) had decreasing LE in the same period. In the highest tertile of baseline LE, 18% (*n* = 177) had increasing LE and 26% (*n* = 254) had decreasing LE from 2011 to 2016. Recognizing that counties could experience increasing or decreasing LE changes regardless of where they started at baseline, LE change was identified to be an indicator that is unique from baseline LE.

### 3.2. LE Change and Modifiable Determinants of Health Indicators

A total of 2943 counties (94% of all counties in the US in 2010) was included in this analysis. Of these, 1239 (42%) counties had no changes in life expectancy, 616 (21%) counties had increasing LE changes, and 1088 (37%) counties had decreasing LE changes from 2011 to 2016 (Table 2). Over half (53%, *n* = 327) of increasing LE counties were in the South and just over a quarter (26%, *n* = 162) were in the Midwest. In contrast, approximately one-third of no-change and decreasing LE counties (33%, *n* = 403; 38%, *n* = 410, respectively) was in the Midwest. No-change counties had a slightly higher mean baseline LE in 2010 (76.6 years, standard deviation (SD): 4.5 years) as compared to increasing (73.7 years, SD: 7.1 years) and decreasing (73.5 years, SD: 6.0) LE counties. Increasing LE counties had a higher proportion of individuals of Hispanic ethnicity (11.8%) as compared to no-change (9.4%) and decreasing LE (7.75%) counties. Notably, increasing LE counties had a substantially higher mean population density (238 people per square mile, SD: 1510) as compared to no-change (101 people per square mile, SD: 231) and decreasing LE (46 people per square mile, SD: 155) counties. The age, sex, and racial category distributions between the counties were similar across LE change categories.

Several modifiable determinants of health were significantly associated with odds of being in increasing LE or decreasing LE counties (Table 3 and Supplementary Materials, Table S2). The odds of being in an increasing (vs. no-change) LE change county decreased by 17% for each increase in the average number of poor physical health days (aOR: 0.825, 95% confidence interval (CI): 0.746, 0.913). We estimated a 10% increase in the odds of experiencing a decreasing (vs. no change) LE for each increase in the average number of poor physical health days (aOR: 1.102, 95% CI: 1.018, 1.193). Higher rates of adult smoking, obesity, unemployment, children in poverty, and single-parent households also increased the odds of experiencing a decrease in LE change, and had similar statistically significant associations with LE change (Table 3). In contrast, binge drinking, motor vehicle crash mortality, and preventable hospital stays were not associated with LE change.

### 3.3. LE Change and COVID-19 Mortality

The analytic dataset included 2200 counties (70% of all US counties in 2019) following the removal of counties with missing or suppressed data for COVID-19 mortality. Of these, 1002 (46%) counties had no change in life expectancy, 779 (35%) counties had decreasing life expectancies, and 419 (19%) counties had increasing life expectancies. Having an increasing LE in 2011–2016, as compared to no change, was significantly associated with a 5% decrease in COVID-19 mortality between 2020 and 2021 (β = 0.953, 95% CI: 0.943, 0.963) (Table 4). Having a decreasing LE was not associated with COVID-19 mortality (β = 0.995; 95% CI: 0.986, 1.004).

## 4. Discussion

LE change is an indicator that may be important for monitoring population health and is unique from the more frequently used measure of single LE. For centuries, public health leaders and scholars have used static life expectancy as a core measure of the health of a population. Although annual trends or changes in LE are monitored [22], a metric of LE change is rarely used at smaller geographic levels to measure the progress or challenges faced by communities in improving a population’s health. The Global Burden of Disease US Health Disparities Collaborators conducted an analysis of county-level trends in LE, showing racial disparities in increases or decreases in LE in different time periods [6]. In places such as the US, where most public and social programs are administered at the local level, county-level LE change may be an important and useful metric to include as part of public health monitoring. Our analysis took the metric of LE change to the next level, showing its association with underlying modifiable determinants of health and also as a predictor of future vulnerability to public health challenges.

In identifying counties that experienced increasing vs. decreasing LE change, we offered a unique metric that augmented the information currently available to local health officials for prioritizing places warranting public health intervention. Maps showing single LE perpetually showed lowest LE areas concentrated in the southeastern US [23]. While recognizing patterns such as these is critical both for focusing interventions as well as identifying root causes of poor population health, being singled out repeatedly in a narrative of poor health risks presenting the issue as “inevitable” and/or “insurmountable”. In contrast, our map depicting LE change at the county level offered a more nuanced picture of health across the US and reflected progress in regions where it may matter most, including in the southeast. There was a mix of no-change, increasing, and decreasing LE counties in all regions, offering opportunities for counties to share interventions, best practices, and lessons learned.

The associations noted between several modifiable determinants of health and LE change were not shocking; these findings presented a validity check for using county-level LE, specifically county-level LE change, as a population health metric. Identifying modifiable determinants of health at the county level that are associated with a core metric of population health offers local health officials opportunities for selecting the most impactful intervention that could result in measurable improvements in the health of their target communities. The County Health Rankings are known and respected as important measures that can guide community-level action to improve health, well-being, and health equity [12]. Linking these to a core health metric provides further guidance for health officials in where they might allocate their limited resources and efforts.

In this study, we showed that counties with increasing LE from 2011 to 2016 had significantly lower COVID-19 mortality compared to no-change counties. This finding offered further evidence that change in LE is a core health metric that can predict the potential for a county to weather future public health emergencies. Not surprisingly, counties with increasing LE, or those that were already succeeding in improving public health and social well-being, were more resilient to the impacts of COVID-19. This finding was consistent with studies that have shown increasing COVID-19 mortality in areas with a lower socioeconomic status and with lower at-birth LE in the US and Europe [24,25]. We did note that decreasing LE counties had a similar COVID-19 mortality to no-change counties. It was not clear why this was the case. We hypothesized that the challenges that heavily affect LE (namely, gun violence and the opioid epidemic) co-occur in counties either less impacted by COVID-19 or more likely to have COVID-19 mortality measurement challenges [26]. Further research should explore this hypothesis.

Our study had several limitations. First, the calculation of small-area life expectancy was challenging, and we did not have the stability of data to conduct this for all the US counties. While we did follow guidance from the National Centers for Health Statistics and other experts, further work is needed to continue to advance this field of estimating LE in all county and age strata. Second, we did not have data for COVID-19 mortality for all the US counties. Particularly during public health emergencies, timely and accurate data compilation is critical, and we encourage future efforts to improve public health surveillance across the US. Finally, clustering techniques, such as group-based trajectory modeling or machine learning, could be considered as alternatives to grouping counties by LE change, rather than using predetermined categorization values [27,28]. This may allow for the accounting of important factors, such as population age, structure, or other unmeasured factors, that contribute to certain counties having similar LE change trajectories.

## 5. Conclusions

We believe our analysis met our objective of demonstrating that change in LE at the county level is a useful metric for tracking public health progress, measuring the impact of public health initiatives, and gauging preparedness and vulnerability for future public health emergencies. In future work, we encourage public health scholars and leaders to include this metric when working to monitor and, ultimately, improve population health, well-being, and equity.

## Figures and Tables

**Table 1:** Comparison of county-level life expectancy (LE) change from 2011 to 2016 and baseline 2010 life expectancy.

County-Level LE Change (2011–2016)	Baseline 2010 Life Expectancy (LE)		
	Tertile 1 (LE = 51–62 Years)	Tertile 2 (LE = 63–74 Years)	Tertile 3 (LE = 75–86 Years )
No change	253 (26%)	436 (44%)	550 (56%)
Increasing	256 (26%)	183 (19%)	177 (18%)
Decreasing	472 (48%)	362 (37%)	254 (26%)

Increasing LE: counties with an annual change in LE slope of ≥0.1; decreasing LE: counties with an annual change in LE slope of ≤−0.1.

**Table 2:** Core sociodemographic characteristics of no-change, increasing life expectancy (LE), and decreasing LE counties (N = 2943 counties).

	No Change (*n* = 1239)	Increasing LE (*n* = 616)	Decreasing LE (*n* = 1088)
Geographic Region (*n*, %)			
Midwest	403 (33%)	162 (26%)	410 (38%)
Northeast	123 (10%)	24 (4%)	69 (6%)
South	565 (46%)	327 (53%)	484 (44%)
West	148 (12%)	103 (17%)	125 (11%)
Baseline LE in 2010 (mean, SD)	76.6 (4.49)	73.7 (7.12)	73.5 (5.95)
Age * (mean, SD)			
Proportion ≤19 years	25.0 (3.17)	25.2 (4.03)	24.6 (3.57)
Proportion >55 years	32.0 (5.68)	31.9 (6.97)	33.7 (5.77)
Proportion Female * (mean, SD)	50.2 (1.85)	49.8 (2.26)	49.8 (2.64)
Proportion Hispanic Ethnicity * (mean, SD)	9.41 (13.3)	11.8 (15.6)	7.75 (13.0)
Proportion Race Category * (mean, SD)			
Non-Hispanic White	76.3 (18.8)	73.0 (21.2)	77.8 (20.5)
Non-Hispanic Black	9.26 (13.5)	8.94 (13.9)	9.59 (16.0)
Non-Hispanic Other	3.02 (5.27)	4.20 (9.06)	3.03 (8.59)
Population Density ** in 2019 (mean, SD)	101 (231)	238 (1510)	46 (155)
Poverty Rate *** (mean, SD)	26.4 (8.09)	26.3 (9.21)	29.1 (8.87)

Increasing LE: counties with an annual change in LE slope of ≥0.1 years; decreasing LE: counties with an annual change in LE slope of ≤−0.1 years. Geographic regions as defined by the US Census Bureau [17]. * Age, sex, race, and ethnicity information was derived from the American Community Survey 2011–2016; the mean of the proportions in each LE change group is reported; ** population density was derived from the US Census Bureau for 2019; the mean population density for each LE change group is reported; *** poverty rate is defined as the proportion of individuals living below 150% of the federal poverty level (FPL), derived from the American Community Survey 2011–2016; the mean of the proportion living below 150% of the FPL in each LE change group is reported.

**Table 3:** Associations between modifiable determinants of health and increasing and decreasing life expectancy (LE) counties.

County Health Ranking Indicator	Odds of Being an Increasing LE County Compared to No Change		Odds of Being a Decreasing LE County Compared to No Change	
	Adjusted Odds Ratio *	95% CI	Adjusted Odds Ratio *	95% CI
Poor or Fair Health	**0.966**	**[0.947, 0.984]**	1.016	[1.000, 1.032]
Poor Physical Health Days	**0.825**	**[0.746, 0.913]**	**1.102**	**[1.018, 1.193]**
Poor Mental Health Days	**0.854**	**[0.768, 0.949]**	1.040	[0.954, 1.133]
Adult Smoking	**0.956**	**[0.937, 0.975]**	**1.019**	**[1.002, 1.037]**
Adult Obesity	**0.923**	**[0.898, 0.95]**	**1.029**	**[1.003, 1.056]**
Binge Drinking	1.014	[0.993, 1.036]	0.992	[0.974, 1.01]
Motor Vehicle Crash Death Rate	0.995	[0.984, 1.005]	**1.010**	**[1.001, 1.019]**
Unemployment	**0.888**	**[0.844, 0.934]**	**1.073**	**[0.939, 0.961]**
Children in Poverty	**0.981**	**[0.969, 0.993]**	**1.018**	**[1.008, 1.029]**
Single-Parent Households	**0.925**	**[0.887, 0.965]**	**1.044**	**[1.008, 1.081]**
Preventable Hospital Stays	0.998	[0.995, 1.001]	**1.003**	**[1.000, 1.005]**
College Degrees	**1.038**	**[1.025, 1.051]**	**0.970**	**[0.958, 0.983]**
Access to Healthy Foods	1.000	[0.995, 1.005]	**0.992**	**[0.988, 0.997]**

Increasing LE: counties with an annual change in LE slope of ≥0.1 years; decreasing LE: counties with an annual change in LE slope of ≤−0.1 years. * Models adjusted for 2010 life expectancy and proportions of the county ≤19 and >55 years of age, bolded values indicate statistical significance (*p* < 0.05).

**Table 4:** Associations between life expectancy change and COVID-19 mortality.

Covariates	Regression Coefficient	95% Confidence Interval
No change in LE	Ref	Ref
Increasing LE	**0.953**	**[0.943, 0.963]**
Decreasing LE	0.995	[0.986, 1.004]
Baseline 2010 LE	**0.928**	**[0.927, 0.930]**
Proportion ≤19 years old in 2019	**1.041**	**[1.039, 1.043]**
Proportion >55 years old in 2019	**1.021**	**[1.019, 1.022]**
Proportion unemployed	**0.985**	**[0.983, 0.987]**
Poor physical health days	**0.965**	**[0.960, 0.971]**
Reference: quintile 1		
Population density—quintile 2	**1.140**	**[1.108, 1.172]**
Population density—quintile 3	**1.307**	**[1.273, 1.342]**
Population density—quintile 4	**1.577**	**[1.538, 1.617]**
Population density—quintile 5	**1.867**	**[1.819, 1.913]**

Increasing LE: counties with an annual change in LE slope of ≥0.1 years; decreasing LE: counties with an annual change in LE slope of ≤−0.1 years. Bolded values indicate statistical significance (*p* < 0.05).

## Data Availability

All data used in this study are publicly available.

## Supplementary Materials

The following supporting information can be downloaded at: https://www.mdpi.com/article/10.3390/ijerph191710672/s1, Table S1: County Health Rankings Indicator Definitions, Table S2: Associations between Modifiable Determinants of Health in 2010 and Life Expectancy Change in 2011–2016.

## References

[1] Roser M., Ortiz-Ospina E., Ritchie H. (2013). Life expectancy. Our World in Data.

[2] Ortaliza J., Ramirez G., Satheeskumar V., Amin K. How does US LIfe Expectancy Compare to Other Countries?. https://www.healthsystemtracker.org/chart-collection/u-s-life-expectancy-compare-countries/#Life%20expectancy%20and%20healthcare%20spending%20per%20capita,%201980-2019%C2%A0.

[3] National Research Council (US) Panel (2011). Explaining Divergent Levels of Longevity in High-Income Countries.

[4] Dwyer-Lindgren L., Bertozzi-Villa A., Stubbs R. (2017). Inequalities in Life Expectancy among US Counties, 1980 to 2014: Temporal Trends and Key Drivers. JAMA Intern. Med..

[5] Mackenbach J.P., Valverde J.R., Bopp M., Bronnum-Hansen H., Deboosere P., Kalediene R., Kovacs K., Leinsalu M., Martikainen P., Menvielle G. (2019). Determinants of inequalities in life expectancy: An international comparative study of eight risk factors. Lancet Public Health.

[6] GBD US Health Disparities Collaborators (2022). Life expectancy by county, race, and ethnicity in the USA, 2000–2019: A systematic analysis of health disparities. Lancet.

[7] Chetty R., Stepner M., Abraham S., Lin S., Scuderi B., Turner N., Bergeron A., Cutler D. (2016). The Association Between Income and Life Expectancy in the United States, 2001–2014. JAMA.

[8] Vaupel J.W., Zhang Z., van Raalte A.A. (2011). Life expectancy and disparity: An international comparison of life table data. BMJ Open.

[9] Montez J.K., Beckfield J., Cooney J.K., Grumbach J.M., Hayward M.D., Koytak H.Z., Woolf S.H., Zajacova A. (2020). US State Policies, Politics, and Life Expectancy. Milbank Q..

[10] Case A., Deaton A. (2015). Rising morbidity and mortality in midlife among white non-Hispanic Americans in the 21st century. Proc. Natl. Acad. Sci. USA.

[11] University of Wisconsin Population Health Institute County Health Rankings & Roadmaps. https://www.countyhealthrankings.org.

[12] Hood C.M., Gennuso K.P., Swain G.R., Catlin B.B. (2016). County Health Rankings: Relationships Between Determinant Factors and Health Outcomes. Am. J. Prev. Med..

[13] Andrasfay T., Goldman N. (2020). Reductions in 2020 US life expectancy due to COVID-19 and the disproportionate impact on the Black and Latino populations. medRxiv.

[14] Arias E., Escobedo L.A., Kennedy J., Fu C., Cisewki J.U.S. (2018). Small-area Life Expectancy Estimates Project: Methodology and Results Summary. Vital Health Stat..

[15] National Center for Health Statistics Restricted-Use Vital Statistics Data. https://www.cdc.gov/nchs/nvss/nvss-restricted-data.htm.

[16] National Center for Health Statistics Provisional COVID-19 Death Counts in the United States by County. https://data.cdc.gov/NCHS/Provisional-COVID-19-Death-Counts-in-the-United-St/kn79-hsxy.

[17] US Census Bureau Geographic Levels. https://www2.census.gov/geo/pdfs/maps-data/maps/reference/us_regdiv.pdf.

[18] Chiang C.L., Robert E. (1984). Lifetable and Its Application.

[19] Karmakar M., Lantz P.M., Tipirneni R. (2021). Association of Social and Demographic Factors With COVID-19 Incidence and Death Rates in the US. JAMA Netw. Open.

[20] Thakur B., Dubey P., Benitez J., Torres J.P., Reddy S., Shokar N., Aung K., Mukherjee D., Dwivedi A.K. (2021). A systematic review and meta-analysis of geographic differences in comorbidities and associated severity and mortality among individuals with COVID-19. Sci. Rep..

[21] Bray I., Gibson A., White J. (2020). Coronavirus disease 2019 mortality: A multivariate ecological analysis in relation to ethnicity, population density, obesity, deprivation and pollution. Public Health.

[22] Woolf S.H., Schoomaker H. (2019). Life Expectancy and Mortality Rates in the United States, 1959–2017. JAMA.

[23] Dobis E.A., Stephens H.M., Skidmore M., Goetz S.J. (2020). Explaining the spatial variation in American life expectancy. Soc. Sci. Med..

[24] Dukhovnov D., Barbieri M. (2022). County-level socio-economic disparities in COVID-19 mortality in the USA. Int. J. Epidemiol..

[25] Wang X.Q., Song G., Yang Z., Chen R.J., Zheng Y.L., Hu H.Y., Su X., Chen P.J. (2020). Association between ageing population, median age, life expectancy and mortality in coronavirus disease (COVID-19). Aging.

[26] United States Government Accountability Office (2020). COVID-19: Data Quality and Considerations for Modeling and Analysis.

[27] Baltrus P., Malhotra K., Rust G., Levine R., Li C., Gaglioti A.H. (2019). Identifying County-Level All-Cause Mortality Rate Trajectories and Their Spatial Distribution Across the United States. Prev. Chronic Dis..

[28] Meshram S.S. Comparative Analysis of LIfe Expectancy between Developed and Developing Countries using Machine Learning. Proceedings of the IEEE Bombay Section Signature Conference (IBSSC).

