# Supplementary material for: County-Level Life Expectancy Change: A Novel Metric for Monitoring Public Health
Source: Int J Environ Res Public Health. 2022 Aug 27;19(17):10672. doi: 10.3390/ijerph191710672 (PMC9517827; doi:10.3390/ijerph191710672)
Supplement: Supplementary file 1 [file ijerph-19-10672-s001.zip › ijerph-1858068-supplementary.pdf]

Supplemental Table S1: County Health Rankings Indicator Definitions

County Health Rankings Indicator	Definition
Premature Death	Years of potential life lost before age 75 (YPLL-75) rate
Poor or Fair Health	Percent of adults reporting fair or poor health
Poor Physical Health Days	Mean physically unhealthy days/month for adult
Poor Mental Health Days	Mean mentally unhealthy days/month for adults
Low Birthweight	Percent of live births with low birthweight (< 2500 grams)
Adult Smoking	Percent of adults that report smoking at least 100 cigarettes and that they currently smoke
Adult Obesity	Percent of adults that report a BMI ≥ 30
Binge Drinking	Percent of adults that report binge drinking in the past 30 days
Motor Vehicle Crash Death Rate	MV deaths per 100K population (crude rate)
Chlamydia Rate	Chlamydia rate per 100K population
Teen Birth Rate	Teen birth rate per 1,000 female population, ages 15–19
Uninsured Adults	Percent of population < age 65 without health insurance
Unemployment	Percent of population age 16+ unemployed but seeking work
Children in Poverty	Percent of children in poverty
Income Inequality	Gini coefficient of income inequality
Inadequate Social Support	Percent of adults without social/emotional support
Single Parent Households	Percent of all households that are single-parent households
Violent Crime Rate	Violent crime rate per 100K population
Homicide Rate	Homicide death rate per 100K population (age-adjusted)
Air Pollution Particulate Matter Days	Annual number of unhealthy air quality days due to fine particulate matter
Air Pollution Ozone Days	Annual number of unhealthy air quality days due to ozone
Liquor Store Density	Number of liquor stores per 10K population
Primary Care Provider Rate	Primary care provider rate per 100K
Preventable Hospital Stays	Hospitalization rate for ambulatory-care sensitive conditions per 1,000 Medicare enrollees
Diabetic Screening	Percent of diabetic Medicare enrollees that receive HbA1c screening
Hospice Use	Percent of chronically ill Medicare enrollees in hospice care in last 6 months of life
High School Graduation	Averaged freshman graduation rate (Percent of ninth grade cohort that graduates in 4 years)
College Degrees	Percent of population age 25+ with 4-year college degree or higher
Access to Healthy Foods	Percent of zip codes in county with healthy food outlets

Supplemental Table S2: Associations between Modifiable Determinants of Health in 2010 and Life Expectancy Change in 2011 - 2016

County Health Rankings Indicator	Odds of being an Increasing LE county compared to No Change		Odds of being a Decreasing LE county compared to No Change	
	**Odds Ratio**	**95% CI**	**Odds Ratio**	**95% CI**
Premature Death	**1.000**	**[1.000, 1.000]**	1.000	[1.000, 1.000]
Poor or Fair Health	**0.966**	**[0.947, 0.984]**	1.016	[1.000, 1.032]
Poor Physical Health Days	**0.825**	**[0.746, 0.913]**	**1.102**	**[1.018, 1.193]**
Poor Mental Health Days	**0.854**	**[0.768, 0.949]**	1.040	[0.954, 1.133]
Low Birthweight	**0.909**	**[0.858, 0.962]**	1.004	[0.957, 1.054]
Adult Smoking	**0.956**	**[0.937, 0.975]**	**1.019**	**[1.002, 1.037]**
Adult Obesity	**0.923**	**[0.898, 0.95]**	**1.029**	**[1.003, 1.056]**
Binge Drinking	1.014	[0.993, 1.036]	0.992	[0.974, 1.01]
Motor Vehicle Crash Death Rate	0.995	[0.984, 1.005]	**1.010**	**[1.001, 1.019]**
Chlamydia Rate	**0.999**	**[0.999, 1.000]**	1.000	[1.000, 1.000]
Teen Birth Rate	**0.991**	**[0.986, 0.996]**	1.003	[0.999, 1.008]
Uninsured Adults	**1.050**	**[1.031, 1.07]**	0.987	[0.971, 1.004]
Unemployment	**0.879**	**[0.834, 0.927]**	**1.050**	**[1.009, 1.094]**
Children in Poverty	**0.981**	**[0.969, 0.993]**	**1.018**	**[1.008, 1.029]**
Income Inequality	1.002	[0.974, 1.03]	0.991	[0.967, 1.016]
Inadequate Social Support	0.984	[0.962, 1.008]	1.008	[0.988, 1.028]
Single Parent Households	**0.925**	**[0.887, 0.965]**	**1.044**	**[1.008, 1.081]**
Violent Crime Rate	**0.999**	**[0.998, 0.999]**	1.000	[0.999, 1.000]
Homicide Rate	0.970	[0.936, 1.005]	1.020	[0.992, 1.048]
Air Pollution Particulate Matter Days	0.981	[0.958, 1.005]	0.984	[0.963, 1.004]
Air Pollution Ozone Days	0.990	[0.975, 1.005]	**0.976**	**[0.961, 0.991]**
Liquor Store Density	1.070	[0.979, 1.17]	1.013	[0.936, 1.097]
Primary Care Provider Rate	**1.003**	**[1.000, 1.005]**	**0.997**	**[0.995, 0.999]**
Preventable Hospital Stays	0.998	[0.995, 1.001]	**1.003**	**[1.000, 1.005]**
Diabetic Screening	0.997	[0.983, 1.01]	0.995	[0.983, 1.007]
Hospice Use	1.003	[0.992, 1.013]	**0.988**	**[0.979, 0.997]**
High School Graduation	**1.009**	**[1.000, 1.017]**	1.001	[0.994, 1.008]
College Degrees	**1.038**	**[1.025, 1.051]**	**0.970**	**[0.958, 0.983]**
Access to Healthy Foods	1.000	[0.995, 1.005]	**0.992**	**[0.988, 0.997]**

Increasing LE: Counties with an annual change in LE slope of ≥0.1 years; Decreasing LE: Counties with an annual change in LE slope of ≤0.1 years.
Bolded values indicate statistical significance at $p<0.05$
